# Carrier Transport and Molecular Displacement Modulated dc Electrical Breakdown of Polypropylene Nanocomposites

**DOI:** 10.3390/polym10111207

**Published:** 2018-10-30

**Authors:** Daomin Min, Chenyu Yan, Rui Mi, Chao Ma, Yin Huang, Shengtao Li, Qingzhou Wu, Zhaoliang Xing

**Affiliations:** 1 State Key Laboratory of Electrical Insulation and Power Equipment, Xi’an Jiaotong University, Xi’an 710049, China; leo-chenyu.yan@stu.xjtu.edu.cn (C.Y.); mr2017@stu.xjtu.edu.cn (R.M.); machao2012@stu.xjtu.edu.cn (C.M.); huangxingyin@stu.xjtu.edu.cn (Y.H.); 2 Institute of Fluid Physics, China Academy of Engineering Physics, Mianyang 621900, China; wuqingzhou@163.com; 3 State Key Laboratory of Advanced Power Transmission Technology, Global Energy Interconnection Research Institute Co. Ltd., Beijing 102209, China; zhaoweimei@sgri.sgcc.com.cn

**Keywords:** carrier transport, polypropylene nanocomposite, molecular chain motion, electrical breakdown, electric energy storage

## Abstract

Dielectric energy storage capacitors have advantages such as ultra-high power density, extremely fast charge and discharge speed, long service lifespan and are significant for pulsed power system, smart power grid, and power electronics. Polypropylene (PP) is one of the most widely used dielectric materials for dielectric energy storage capacitors. It is of interest to investigate how to improve its electrical breakdown strength by nanodoping and the influencing mechanism of nanodoping on the electrical breakdown properties of polymer nanocomposites. PP/Al_2_O_3_ nanocomposite dielectric materials with various weight fraction of nanoparticles are fabricated by melt-blending and hot-pressing methods. Thermally stimulated current, surface potential decay, and dc electrical breakdown experiments show that deep trap properties and associated molecular chain motion are changed by incorporating nanofillers into polymer matrix, resulting in the variations in conductivity and dc electrical breakdown field of nanocomposite dielectrics. Then, a charge transport and molecular displacement modulated electrical breakdown model is utilized to simulate the dc electrical breakdown behavior. It is found that isolated interfacial regions formed in nanocomposite dielectrics at relatively low loadings reduce the effective carrier mobility and strengthen the interaction between molecular chains, hindering the transport of charges and the displacement of molecular chains with occupied deep traps. Accordingly, the electrical breakdown strength is enhanced at relatively low loadings. Interfacial regions may overlap in nanocomposite dielectrics at relatively high loadings so that the effective carrier mobility decreases and the interaction between molecular chains may be weakened. Consequently, the molecular motion is accelerated by electric force, leading to the decrease in electrical breakdown strength. The experiments and simulations reveals that the influence of nanodoping on dc electrical breakdown properties may origin from the changes in the charge transport and molecular displacement characteristics caused by interfacial regions in nanocomposite dielectrics.

## 1. Introduction

With the continuous increase in energy consumption and the gradually deterioration of environment caused by using fossil energy, sustainable energy technology becomes more and more important for the energy transformation [1,2]. Energy storage technology is one key issue for the integration of renewable energies such as wind energy and solar energy to power grid [3]. Dielectric energy storage capacitor has the advantages of ultra-high power density, extremely fast charge and discharge speed, environment friendly, long service lifespan, and low manufacturing cost [4], and has a great potential for the application in smart power grid, pulsed power system, and power electronics [4,5]. Capacitors store and release electric energy via polarization and depolarization of dielectric materials, which has no mass diffusion process. Consequently, capacitors can store and release electric energy in a very short period, which means they can provide high power density for power supply system to tune the peak power output [4,5,6]. Since no chemical reactions involve in the storage and release of electric energy and dielectric materials such as polypropylene (PP), polyester, and polyimide maintain high electrical breakdown strength at relatively high temperatures, dielectric energy storage capacitor has long cycle life and can operate in high temperature circumstance [5,6,7]. However, the energy density of dielectric energy storage capacitors is very low, limiting its application and miniaturization. It is necessary to develop dielectric materials with high electrical breakdown strength and high dielectric constant to enhance the energy density of dielectric energy storage capacitors [4,5].

Incorporating a small amount of nanofillers into polymers to fabricate nanocomposite materials is an effective approach to enhance their electrical breakdown strength [6,8]. Before the incorporation of nanofillers into polymers, surface modification of nanofillers and/or chemical grafting modification of polymers are usually adopted to improve the bonding strength between nanofillers and the matrices and the dispersion of nanofillers in the matrices [6,8]. Surface modification of nanofillers can decrease their surface energy and improve their compatibility with matrices. Accordingly, surface modified nanofillers can disperse more homogeneously in polymer matrices, and nanocomposites can obtain better electrical performance than neat polymers. The surfaces of MgO, TiO_2_, Al_2_O_3_, and ZnO nanoparticles were modified by γ-methacryloxypropyltrimethoxysilane firstly, and then were separately doped in PP matrix to fabricate nanocomposite dielectric materials [9]. Experimental results revealed that electrical breakdown strengths of all PP nanocomposites increase firstly and then decrease. PP based TiO_2_, Al_2_O_3_, and ZnO nanocomposite dielectric materials show maximum electrical breakdown strengths at nanofiller loading of 1 wt %, while PP/MgO nanocomposite samples has the maximum value at 3 wt % [9]. It was also found that the PP/MgO nanocomposite materials with a loading of 1 wt % has a remarkable enhancement of dc electrical breakdown strength compared with PP matrix [10]. In addition, it was found that PP based silica nanocomposite dielectrics have higher electrical breakdown strengths under both dc and ac ramp voltages [11]. Incorporation of nanoparticles can enhance the electrical breakdown strength of polymer matrix, but the influencing mechanism of nanodoping on electrical breakdown strength remains to be investigated.

Interfacial region models for polymer nanocomposite dielectric materials were proposed based on mesoscopic structure, morphology, molecular motion, crystallization, and electrical properties, and so forth, demonstrating the direction to unravel the mechanism that how nanodoping enhance the electrical breakdown strength of polymer nanocomposites [8,12,13,14,15,16]. For example, Lewis proposed an electric double layer model based on colloidal theory. When nanoparticle loading is relatively low, barriers in electric double layers impede the migration of carriers, leading to the decrease in conductivity and enhancement in electrical breakdown strength. With a further increase in nanoparticle loading, overlapping of interfacial regions appears. This will assist to form conduction path within the bulk of nanocomposites under the applied voltage and accelerate carrier transport, resulting in the increase in conductivity and the decrease in electrical breakdown strength [12,13]. In addition, Tanaka et al. proposed a multi-core model and suggested that interfacial region consists of bonded, bound, and loose layers. Vast deep traps exist in the regions of bonded and bound layers, while shallow traps mainly exist in the region of loose layer [15,16]. Furthermore, Li et al. [14] introduced a multi-region model, which indicated that interfacial region is composed of bonded, transitional, and normal regions. Deep traps mainly are in the bonded and transitional regions, while shallow traps are mainly located in normal region. It can be inferred from the multi-core model [15,16] and multi-region model [14] that when a small amount of nanoparticles are doped, deep traps have dominant effect on electrical breakdown property of nanocomposites. This leads to the decrease in density and energy of carriers as well as conductivity, resulting in an enhancement in electrical breakdown strength. When nanoparticle loading further increases, interfacial regions will start to overlap and the effect brought by shallow traps will be strengthened, resulting in a decrease in electrical breakdown strength.

The increase in energy and/or density of deep traps can suppress charge injection from electrodes. Consequently, the accumulation of space charges is reduced and the distortion of electric field is mitigated, leading to an increase in electrical breakdown strength [8,15,16,17,18]. When an electric field of 60 kV mm^−1^ is applied on the sample for 30 min at room temperature, homo space charges accumulate at the regions near electrodes. With an increase in nanofiller loading, accumulation of homo space charges shows a decrease and then is followed by an increase, and distortion of electric field has the similar profile. Thus dc electric field increases firstly and then decreases [19]. Additionally, for PP/Fullerene nanocomposites, when applying an electric field of 60 kV mm^−1^ on the samples, pulsed electro-acoustic (PEA) results indicated that for the sample with a loading of 1 wt %, accumulation of homo space charges within the sample is effectively suppressed and distortion of electric field is mitigated. The sample with this nanofiller loading has higher dc electrical breakdown strength compared with polymeric matrix [20].

In order to investigate the influencing mechanism of nano-doping on electrical breakdown properties of PP nanocomposite dielectric material, PP/Al_2_O_3_ nanocomposites were fabricated and their trap distribution and dc electrical breakdown properties were measured. The relation between deep trap properties and dc electrical breakdown strength was analyzed. Then, a model considering carrier transport and molecular displacement dynamics was utilized to simulate the dc electrical breakdown behavior of PP nanocomposite dielectric materials.

## 2. Materials and Methods

### 2.1. Fabrication of PP/Al_2_O_3_ Nanocomposite Materials

Semi-crystalline granular PP homopolymer (Borclean^TM^ HC312BF) about 3 mm in diameter was purchased from Borealis, Vienna, Austria. The PP homopolymer has a melt flow rate of 4.4 g/10 min at 190 °C/5 kg, an isotactic index of 96%, and a melting temperature of 161–165 °C. Nano-Al_2_O_3_ particles about 30 nm in diameter, with purity beyond 99.99% was supplied by Beijing DK nano technology Co., Ltd., China. In addition, γ-methacryloxy propyltrimethoxy silane coupling agent (KH570) was also supplied by Beijing DK nano technology Co., Ltd., China.

Soft agglomeration and hard agglomeration may be formed in inorganic nanparticles in powder state induced by physical forces such as electrostatic force and Van Der Waals force and by bond interaction or chemical effect existing among the particles, respectively. Accordingly, nanoparticles were dried in a vacuum oven at 100 °C for 12 h to eliminate humidity inside. Then, nanoparticles were mixed with ethanol and the mixture was sonicated by an Ultrasonic Oscillator (Xinyi-2400F, Ningbo Xinyi Ultrasonic Instrument Co., Ltd., Ningbo, China) at a frequency of 40 kHz and a power of 100 W for 30 min around room temperature. After that, the mixture was processed by an Ultra-high Pressure Homogenizer (SPCH-18, Stansted Fluid Power, Stansted, UK) at a pressure of 40 MPa at room temperature for 5 times. The treatments by Ultrasonic Oscillator and Ultra-high Pressure Homogenizer can eliminate the agglomerations of nanoparticles.

Surface modification on nanoparticles by γ-methacryloxy propyltrimethoxy silane coupling agent can reduce the electrostatic force and increase the compatibilisation and coupling interaction between nanparticles and PP matrix, which will lead to a more homogeneous dispersion in the polymer matrix. Firstly, 5 g γ-methacryloxy propyltrimethoxy silane coupling agent was hydrolyzed in 10 g deionized water. Hydrolysis reaction of coupling agent was carried out under 80 °C with a magnetic stirrer until the solution was observed transparent. When the solution was transparent, the hydrolysis reaction was nearly complete. Then, the homogenized mixture was were mixed the hydrolyzed solution of γ-methacryloxy propyltrimethoxy silane coupling agent. The reaction between nanoparticles and coupling agent was carried out under 80 °C, stirring by a Magnetic Stir for 12 h. Thirdly, the mixture was centrifuged by a centrifugal machine (HC-3514, Anhui USTC Zonkia Scientific Instruments Co., Ltd., Hefei, China) at speed of 4000 rmin^−1^ for 10 min, and then ultrasonic processed for 20 min. The cleaning process was repeated for 5 times so that extra coupling agent, reaction accessory product, and impurities can be removed. Subsequently, the mixture was dried under 100 °C for 12 h and grinded prior to use.

After the surface modification by γ-methacryloxy propyltrimethoxy silane coupling agent, nanoparticles were introduced into molten PP matrix and dispersed inside by using a Torque Rheometer (RC-90, HAAKE PolyLab QC, Waltham, MA, USA). During the melt blending process, the temperature of Torque Rheometer was set as 175 °C and rotation speed was 40 r/min. The melting blend time was 10 min. A series of PP nanocomposites with various weight fractions of nano-Al_2_O_3_ particles, namely, 0.5 wt %, 1 wt % and 2 wt % were prepared. In addition, to ensure the consistency of samples, neat PP samples were also prepared by the same melting method.

Then, sheet samples were prepared through hot-pressing by Plate Vulcanizing machine (YT-LH-20B, Yitong Technology Co., Ltd., Shenzhen, China). After preheating for 15 min without pressure, samples were pressed with a mould under a pressure of 10 MPa at 240 °C for 10 min. Finally, water-cooling process without external pressure was carried out until the samples cool down to room temperature. The average thickness of samples is about 100 μm with a standard deviation of 3.5 μm. The densities are 0.926, 0.930, 0.937, and 0.946 g/m^3^ for the samples with loadings of 0 wt %, 0.5 wt %, 1 wt % and 2 wt %, respectively.

### 2.2. Characterization of PP/Al_2_O_3_ Nanocomposite Materials

The dispersion quality of Al_2_O_3_ nanoparticles in PP matrix was investigated by a Scanning Electron Microscope (SEM, GeminiSEM 500, Carl Zeiss AG, Oberkochen, Germany). Dielectric materials were freeze fractured in liquid nitrogen. Then, the cross section of the freeze fractured samples were sputtered with Au by an Ion Sputter (MC1000, Hitachi, Ltd., Tokyo, Japan) as the samples are non-conductive dielectrics. The morphology of fracture surfaces of nanocomposite materials was observed by the SEM with an accelerating voltage of 5 kV. 

Thermally stimulated depolarization current (TSDC, Concept 90, Novocontrol technologies, Frankfurt, Germany) measurement was carried out on PP/Al_2_O_3_ nanocomposite samples to study their trap distribution properties. Before the TSDC measurements, Au electrodes with diameter of 30 mm were sputtered on both sides of samples. Then the sample was put into the vacuum chamber of TSDC apparatus. The sample was heated to 80 °C and then was polarized under an external applied voltage of 250 V for 20 min. After the polarization, the sample was cooled down to −40 °C by liquid nitrogen and then short-circuited for 3 min. Afterwards, the sample was heated with a ramping rate of 2 °C·min^−1^ up to 150 °C. At the same time, depolarization current was measured by a picoammeter (6514B, Keithley, Beaverton, OR, USA).

The conductivity property of nanocomposites were investigated by surface potential decay (SPD) measurement. The experimental process can be divided into surface potential built-up and decay processes. During the surface potential built-up process, a dielectric sample was charged by corona produced by a needle-grid electrode system. The needle and grid electrodes were applied voltages of −13 kV and −3 kV, respectively. Each sample was charged for 3 min. While negative voltage is applied to the needle electrode, high energy electrons emitted from the needle electrode will migrate along electric field and then collide with neutral molecules in the air, generating secondary electrons and positive ions. Then, these electrons can combine with neutral molecules to form negative ions. Under the effect of negative voltage on gird electrode, negative ions move towards dielectric material and transfer electrons to the sample surface. After charging process, the needle-grid electrode system was moved away and an electrostatic voltmeter probe (3453ST, Trek, Lockport, NY, USA) was moved to the charging position. Surface potentials of samples were measured by a high-voltage electrostatic voltmeter (P0865, Trek, Lockport, NY, USA) for 150 min, and recorded by a Labview^®^ program. All the SPD experiments were carried out at a temperature of 33 °C and a humidity of 49 RH%. 

DC electrical breakdown fields of PP/Al_2_O_3_ nanocomposites were measured by a computer control voltage breakdown testing apparatus (HJC-100kV, Huayang instrumentation Co., Ltd., Yangzhong, China). Sphere-sphere copper electrodes with a diameter of 25 mm were used in dc electrical breakdown experiments. The experiments were performed in transformer oil (25#, China National Petroleum Corporation, Karamay, China) at room temperature (around 33 °C). A ramp dc voltage with a rising rate of 2 kV s^−1^ was applied across the samples until breakdown occurs. The electrical breakdown voltage when the failure took place was recorded by a computer program. For each sample, dc electrical breakdown experiments were repeated for fifteen times.

## 3. Experimental Results

### 3.1. Morphology of PP/Al_2_O_3_ Nanocomposite Materials

**Figure 1:**
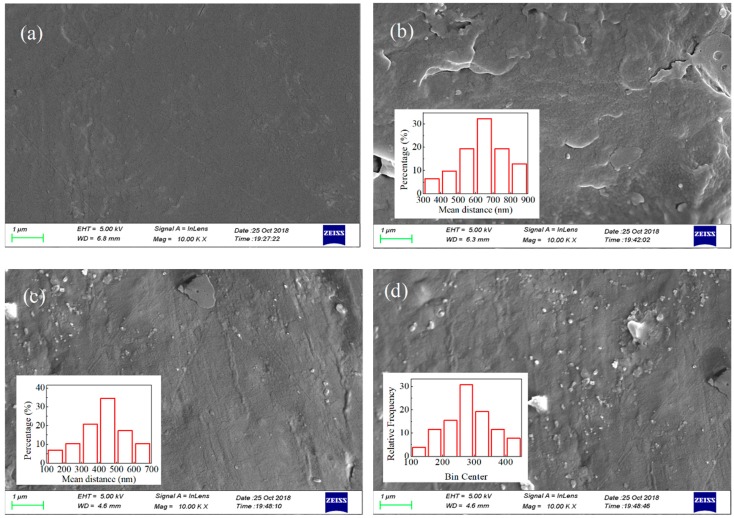
Morphology images of fracture surfaces of PP/Al_2_O_3_ nanocomposites at various nanofiller loadings, (**a**) 0 wt %, (**b**) 0.5 wt %, (**c**) 1.0 wt %, and (**d**) 2.0 wt %, observed by a high energy SEM.

Figure 1 depicts the morphology images of fractured surfaces of PP/Al_2_O_3_ nanocomposite samples with various nanofiller loadings. It is observed from Figure 1a that a monodirectional force trace like a river can be obviously seen on the fracture surface of the sample, and the regions of fracture surface are very smooth. Therefore, fractured surface of neat PP sample reveals a brittle fracture. When a small amount of nanofiller is doped, nanofillers are homogeneously dispersed in the matrix and no agglomeration is observed. With a further increase in nanofiller loading, mean distance between nanoparticles is gradually decreased. ImageJ was introduced to numerically demonstrate the nanoparticle dispersion in the matrix. Mean distance between nanofillers obey Gaussian distribution and are with estimated average values of 623, 435, and 278 nm for PP/Al_2_O_3_ samples with loadings of 0.5 wt %, 1 wt %, and 2 wt %, respectively. Results indicate that mean distance between nanoparticles gradually decreases with an increase in nanofiller loading.

### 3.2. Trap Properties for Charge Carriers

**Figure 2:**
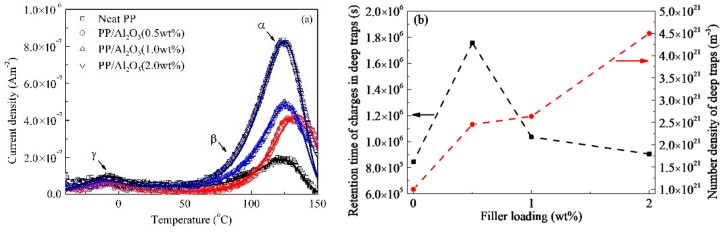
Results of TSDC measurement. (**a**) Comparison of experimental TSDC and fitting results. Symbols represent experimental results and solid lines on symbols are corresponding fitting results calculated by Equation (1). (**b**) Energy and density of deep traps calculated by Equations (1) and (2) as a function of nanofiller loading.

The TSDC experimental results of PP/Al_2_O_3_ nanocomposite dielectric materials are shown in Figure 2a. Two obvious relaxation peaks are observed in TSDC curves for each sample, which are located at temperatures of −10 °C and 125 °C. Another relaxation peak is probably located at around 80 °C. Three peaks from high temperature to low temperature are named as peak α, peak *β*, and peak γ. It is observed obviously that relaxation peaks α and *β* of PP nanocomposite dielectric materials are changed by incorporating different loadings of nanoparticles. For example, peak value of α increases in magnitude with an increase in nanofiller loading. In order to extract the trapping parameters of nanocomposite dielectrics, experimental results were analyzed by TSDC equation [21],

(1) $$j_{\mathrm{TSC}}\left(T\right)=B\exp\left[-\frac{E_{a}}{k_{B}T}-\frac{1}{\beta \tau_{0}}\int_{T_{0}}^{T}\exp\left(-\frac{E_{a}}{k_{B}T}\right)dT\right]$$

Here, *j*_TSC_(*T*) is the TSDC current density in Am^−2^, *B* is an undermined factor in Am^−2^, *E*_a_ is the activation energy of relaxation process in eV, *τ*_0_ is the relaxation time constant in s, *β* is heating rate in K s^−1^, *k*_B_ is the Boltzmann constant, *T*_0_ is the initial temperature of sample at the beginning of heating process in °C, and *T* is the temperature of sample after heating in °C.

Figure 2a shows that fitting curves with three relaxation processes are in a good agreement with TSDC experiments and the three relaxation peaks are separated. Peak α at about 125 °C is possible to correspond to the traps that can capture carriers. Peak *β* at around 83 °C may correspond to the traps that can assist the carrier hopping process in dielectrics. Peak γ with relatively low magnitude is observed at around −10 °C, which may reflect the glass transition process. Traps of peak α indicates the deep traps while those of peak *β* are considered as shallow traps. The energy of deep traps extracted from the TSDC is 0.86 eV for PP/Al_2_O_3_ sample with various nanofiller loadings, Similar results were found by other researchers. The energies of deep traps in polypropylene/propylene ethylene rubber/ZnO ternary nanocomposites samples with different nanofiller loadings are measured to be 0.84–0.87 eV by TSDC method [22]. It was also found that the trap energies of neat PP, polypropylene/propylene-ethylene copolymer, and polypropylene/ethylene-octene copolymer samples were measured to be around 0.87 eV [19]. In addition, through conduction current measurement trap energy of neat block polypropylene was calculated to be around 0.92 eV [23]. The relaxation time constant *τ*_0_ of relaxation process α changes with the loading, which is 5.79 × 10^−9^ s at 0 wt %, 1.20 × 10^−8^ s at 0.5 wt %, 7.10 × 10^−9^ s at 1.0 wt %, and 6.20 × 10^−9^ s at 2.0 wt %. Accordingly, the retention times of deep trapped charges, *τ*_de_ = *τ*_0_exp(*E*_a_/*k*_B_*T*), increases firstly and then decreases with an increase in nanofiller loading and are 8.45 × 10^5^, 1.76 × 10^6^, 1.04 × 10^6^, and 9.04 × 10^5^ s for nanocomposite dielectrics at loadings of 0 wt %, 0.5 wt %, 1.0 wt %, and 2.0 wt %, respectively, as show in Figure 2b.

The density of deep traps corresponds to the peak value of relaxation process α and it can be figured out by considering parameter *B* in Equation (1). It can be described by following equation,

(2) $$N_{T}\left(\xi \right)=N_{T}\left(0\right)B\left(\xi \right)/B\left(0\right)$$

where *ξ* is the nanofiller loading. The deep trap density of neat PP, *N*_T_(0), is assumed to be 1 × 10^21^ m^−3^ [24,25]. In this regard, trap densities are 1.25 × 10^21^, 1.59 × 10^21^, and 2.81 × 10^21^ m^−3^ for PP/Al_2_O_3_ sample with loadings of 0 wt %, 0.5 wt %, 1 wt %, and 2 wt %, respectively. Figure 2b shows the energy and density of deep traps as a function of nanofiller loading. It is indicated that the energy of deep traps increases firstly and then decreases with an increase in nanofiller loading, while the density of deep traps increases monotonically.

### 3.3. DC Conductivity

**Figure 3:**
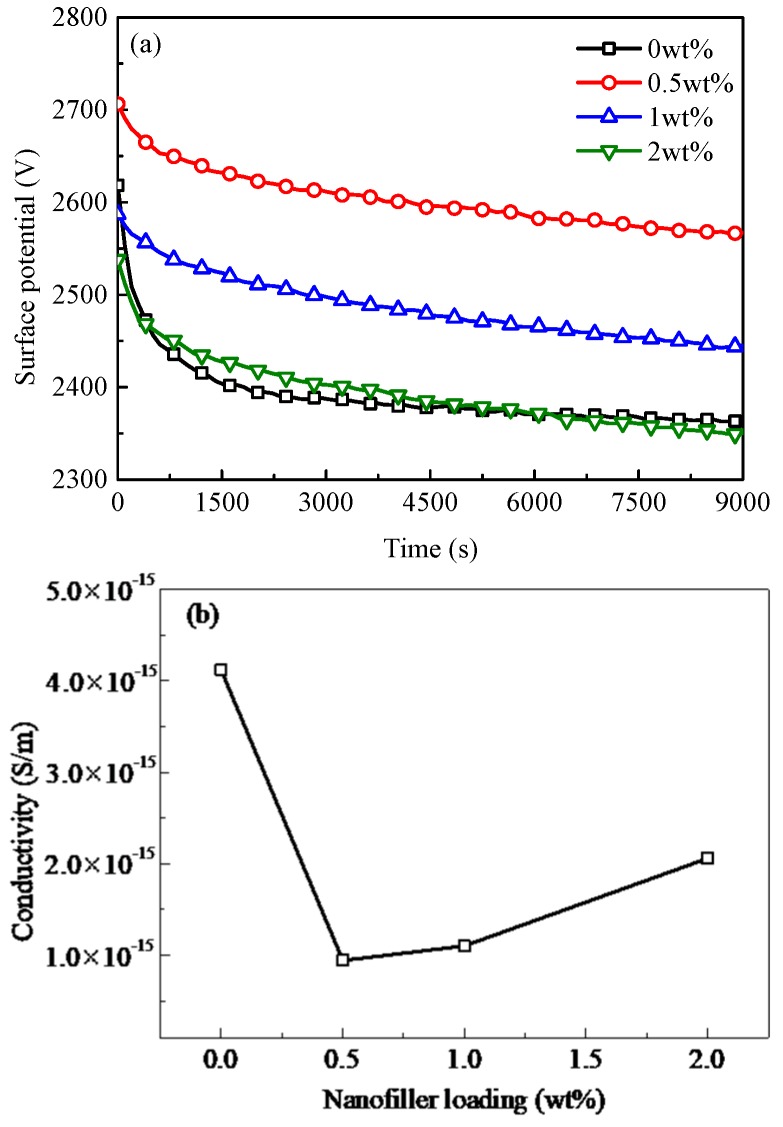
Experimental results of SPD for PP/Al_2_O_3_ nanocomposites with different nanofiller loadings (**a**) and conductivity calculated by Equation (3) as a function of nanofiller loading (**b**).

Figure 3a shows SPD experimental results of PP/Al_2_O_3_ nanocomposites. Potential decay rate increases firstly and then is followed by a decrease with an increase in nanofiller loading. For a film polymeric dielectric, the decay of surface potential is dominated by the migration of charge carriers through the material into grounded electrode, so it reflects similar changes in bulk conductivity [26,27,28,29]. The bulk conductivity of nanocomposites can be calculated from surface potential measurement results via the following equation [30]:

(3) $$\gamma \left(\phi_{s}\right)=-\frac{d\phi_{s}\left(t\right)}{dt}\frac{\varepsilon_{0}\varepsilon_{r}}{\phi_{s}\left(t\right)}$$

Here, *φ*_s_ is surface potential in V, *t* is the decay time in s, *ε*_0_ is the vacuum permittivity in Fm^−1^, *ε*_r_ is the relative permittivity of nanocomposites, and *γ* is the bulk conductivity in Sm^−1^.

Figure 3b depicts bulk conductivity of nanocomposite dielectrics as a function of nanofiller loading. The bulk conductivity of neat PP sample is about 4.12 × 10^−15^ Sm^−1^. In addition, conductivity has a decrease and then is followed by a monotonic increase with an increase in nanofiller loading. Minimum conductivity reaches 9.43 × 10^−16^ Sm^−1^ at the nanofiller loading of 0.5 wt %. This trend in PP nanocomposites was also observed by other researchers [9].

### 3.4. DC Electrical Breakdown Strength of PP Nanocomposites

Electrical breakdown property is generally evaluated by Weibull-distribution. In this work, two-parameter Weibull-distribution can be expressed as [31],

(4) $$P_{i}=1-\exp\left[-{\left(F_{\mathrm{bi}}/\alpha \right)}^{\beta }\right],\;\left(i=1,\;2,\;\ldots ,\;n\right)$$

Here, *i* is the electrical breakdown strength array sorted from the small to large, *n* represents the testing time for the same sample, *P*_i_ is cumulative electrical breakdown probability of the data numbered with *i*, which is related to *i* and *n*, e.g., *P*_i_ = (*i* − 0.44)/(*n* + 0.25) [31]. Furthermore, *F*_b_ is the experimental result of dc electrical breakdown field in kV mm^−1^, *α* is characteristic electrical breakdown strength with accumulative probability of 63.2%, and *β* is shape distribution parameter. 

**Figure 4:**
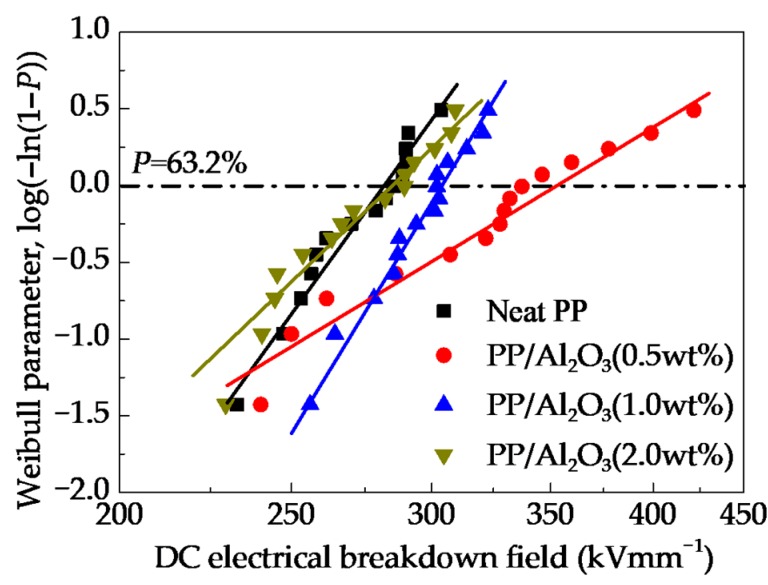
Two-parameter Weibull-distributions of dc electrical breakdown field for PP/Al_2_O_3_ nanocomposites with various nanofiller loadings. Symbols represent experimental results of dc electrical breakdown and solid lines are calculated results using Equation (4).

Figure 4 shows the Weibull-distributions of dc electrical breakdown field for PP/Al_2_O_3_ nanocomposites at various nanofiller loadings. Fitted by Equation (4), characteristic electrical breakdown fields *F*_b_ were obtained as shown in Figure 4. DC electrical breakdown field has an increase then is followed by a decrease with an increase in nanofiller loading. The characteristic electrical breakdown fields are 282, 353, 304 and 285 kV mm^−1^ for PP/Al_2_O_3_ nanocomposites with loading of 0 wt %, 0.5 wt %, 1 wt %, and 2 wt %, respectively. The maximum value of characteristic electrical breakdown field appears around 0.5 wt %. The dc electrical breakdown field of PP/Al_2_O_3_ nanocomposite material with the loading of 0.5 wt % is 25.2% larger than that of neat PP.

### 3.5. Influence of Trap Properties on dc Conductivity and Electrical Breakdown Strength

**Figure 5:**
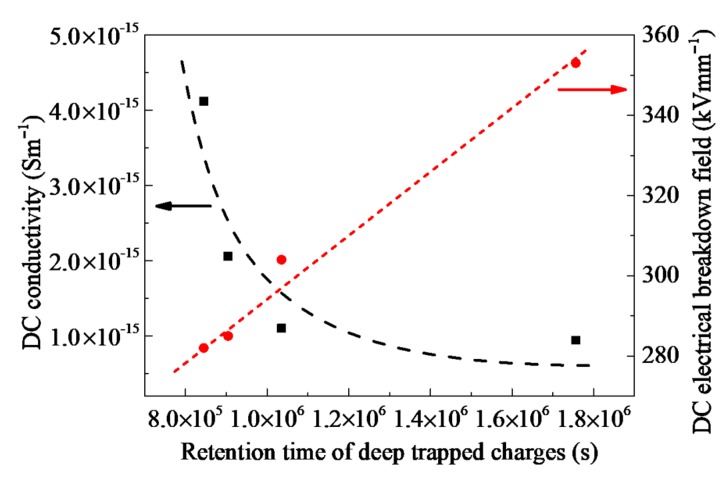
DC conductivity and dc electrical breakdown field of PP/Al_2_O_3_ nanocomposites as a function of the retention time of deep trapped charges. Symbols represent the experimental results, while dash curves represent the potential types of relations.

Figure 5 shows dc conductivity and electrical breakdown strength of PP/Al_2_O_3_ nanocomposites as a function of the retention time of deep trapped charges. Conductivity *γ* decreases with an increase in the retention time of deep trapped charges *τ*_de_. According to Arrhenius equation, *γ* = *γ*_0_*τ*_tr_/(*τ*_tr_ + *τ*_de_), the conductivity is proportional to the reciprocal *τ*_de_, when *τ*_tr_ is much smaller than *τ*_de_ [31]. Here *γ*_0_ is a prefactor of exponential equation and *τ*_tr_ is the mean time period of mobile charge carriers between two trapping events in s. The retention time of trapped charges at deep trap centers is determined by trap energy and temperature, *τ*_de_ = *τ*_0_exp(*E*_T_/*k*_B_*T*) [31,32]. Mobile charges injected from electrodes can be captured by deep traps and the retention time of trapped carriers at deeper traps is longer. This will lead to lower conductivity. As an increase in the retention time of trapped carriers, more charges are trapped at the areas near the interfaces between dielectric material and its electrodes, suppressing the accumulation of space charges and mitigating the distortion of electric field. Accordingly, electrical breakdown strength increases with an increase in the retention time of trapped carriers.

Experimental results measured by PEA method indicated that under high electric fields homo space charges can accumulate in the bulk of PP dielectric material [9,19,20,23]. The accumulation of space charges results in the distortion of electric field. The accumulation of homo space charges leads to a decrease in electric fields near the interfaces between the dielectric material and its electrodes and an increase in electric fields in the middle of bulk [9,19,20,23]. It was also reported that the accumulation of space charges increases and electric field distortion is much severer with an increase in the applied electric field [20]. Space charge accumulation and electric field distortion experiments at ultra-high external electric fields demonstrated that electrical breakdown was initiated when the maximum distorted electric field exceeds a threshold value [17,33]. In addition, incorporation of a slight amount nanofillers can suppress the accumulation of space charges in the bulk of material to some extent and electric field distortion can also be mitigated. The comparison of space charge accumulation and dc electrical breakdown results between polyethylene nanocomposites with relatively low nanofiller loadings and neat polyethylene indicated that carrier transport is closely relating to the electrical breakdown property of dielectric material [17,33]. However, excessive increase in nanofiller loading can result in an increase in accumulation of space charges and reinforcement in electric field distortion [8,14]. When nanofiller loading exceeds a threshold value, accumulation of space charges can even be larger than that in neat sample [9]. The quantitative relation between nanodoping and electrical breakdown strength is not fully understood in PP nanocomposites. In the following part, a carrier transport and molecular displacement modulated electrical breakdown model will be used to investigate the influencing mechanism of incorporating nanoparticles into polymers on dc electrical breakdown properties.

## 4. Modelling Analysis of dc Electrical Breakdown of PP Nanocomposites

### 4.1. Carrier Transport and Molecular Displacement Modulated Electrical Breakdown Model

**Figure 6:**
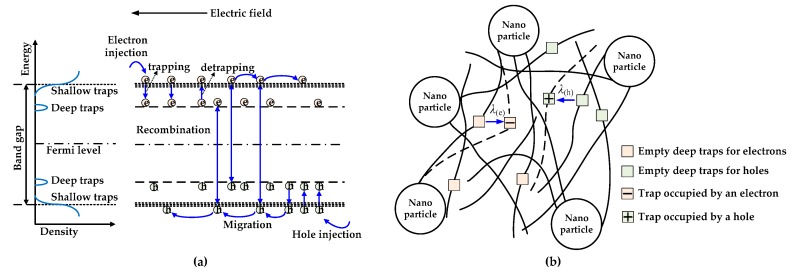
Schematic of carrier transport and molecular displacement modulated electrical breakdown model for polymer nanocomposite dielectric materials. (**a**) Charge injection, migration, trapping, detrapping and recombination dynamics in polymer nanocomposites under external electric field. (**b**) Molecular chains with occupied deep traps move toward electrodes driven by Coulomb force.

As shown in Figure 6, a carrier transport and molecular displacement modulated electrical breakdown (CTMD) model considering five processes, namely charge injection, charge migration, charge trapping/detrapping, recombination dynamics, and molecular chain motion is introduced to investigate the electrical breakdown property of PP/Al_2_O_3_ nanocomposite dielectric materials [18,34,35,36,37]. The dielectric materials are clamped by two electrodes, and the cathode and anode are at the positions of *x* = 0 and *x* = *d*, respectively. Here, *d* is the thickness of sample. A ramp voltage with a rising rate of *k*_ramp_ is applied to the material, and the external voltage is the product of ramping rate and elapsed time of ramping voltage *t*, which is also the boundary condition of the CTMD model.

(5) $$V_{\mathrm{appl}}\left(t\right)=k_{\mathrm{ramp}}t$$

Here, *V*_appl_ is external applied voltage in V, and *k*_ramp_ is the ramp rate of voltage source in V s^−1^.

Charges in the bulk can be categorized to two types, mobile charges that move in shallow traps and trapped charges that are captured by deep traps. Charge carriers captured by deep traps will accumulate space charges in dielectric materials. The accumulation of space charges can result in the local distortion of electric field *F*, which is the minus gradient of electric potential *ϕ*, namely, *F* = −∇*ϕ*. The relation between space charges and electric potential can be solved by Poisson’s equation [31],

(6) $$\frac{\partial^{2}\phi \left(x,t\right)}{\partial x^{2}}=-\frac{e\left[-n_{\mathrm{free}\left(e\right)}\left(x,t\right)-n_{\mathrm{trap}\left(e\right)}\left(x,t\right)+n_{\mathrm{free}\left(h\right)}\left(x,t\right)+n_{\mathrm{trap}\left(h\right)}\left(x,t\right)\right]}{\varepsilon_{0}\varepsilon_{r}}$$

Here, *n*_free(e)_ and *n*_trap(e)_ are the number densities of negative mobile and negative trapped charges in m^−3^, while *n*_free(h)_ and *n*_trap(h)_ are the number densities of positive mobile and positive trapped charges in m^−3^ and *e* is the elementary charge in C. The boundary conditions are *φ*(0) = 0 V and *φ*(*d*,*t*) = *V*_appl_(*t*).

Negative and positive charge carriers are assumed to be injected from the cathode and anode, respectively, into nanocomposite dielectric materials by modified Schottky thermionic emission [38]. The current densities of carrier injections are determined by effective injection barriers between the dielectric material and its electrodes and electric fields at the interfaces [38].

(7) $$j_{in\left(e\right)}\left(t\right)=\frac{2\psi^{2}e^{2}N_{C}{}^{1/3}\upsilon_{0}F\left(0,t\right)}{k_{B}T}\exp\left(-\frac{E_{in\left(e\right)}}{k_{B}T}\right)\exp f^{1/2}$$

(8) $$j_{in\left(h\right)}\left(t\right)=\frac{2\psi^{2}e^{2}N_{V}{}^{1/3}\upsilon_{0}F\left(d,t\right)}{k_{B}T}\exp\left(-\frac{E_{in\left(h\right)}}{k_{B}T}\right)\exp f^{1/2}$$

Here, *j*_in(e)_ and *j*_in(h)_ represent the current densities caused by negative charges injected from the cathode and positive charges injected from the anode into the nanocomposite material respectively in A m^−2^, *E*_in(e)_ and *E*_in(h)_ are the effective injection barriers for electrons and holes respectively in eV, *N*_C_ and *N*_V_ are the density of states in conduction band and valence band respectively, *F*(0*, t*) and *F*(*d, t*) are the electric fields at the interfaces of *x* = 0 and *x* = *d* respectively, and *υ*_0_ is the attempt to escape frequency in s^−1^, which is the reciprocal of relaxation time constant of trapped charges, *υ*_0_ = 1/*τ*_0_. Furthermore, the reduced electric field *f* is determined by coulomb radius, electric field, and temperature, which is *f* = *eFr*_c_/*k*_B_*T*. Here *r_c_* is the coulomb radius in m, *r*_c_ *= e^2^/4πε*_0_*ε*_r_*k*_B_*T.* Then, the field-dependent parameter *ψ* can be obtained by *ψ*(*f*) *= f*^−1^ *+ f*^−1/2^ *− f*^−1^(1 *+* 2*f*^1/2^)^1/2^ [38].

As demonstrated in Figure 6a, under the application of electric field, the injected electrons and holes can hop between shallow traps toward the anode and cathode, respectively. The migration of charge carriers can form conduction currents in the bulk of dielectric martials [18,24,34,35,36],

(9) $$j_{c\left(e\right)}(x,t)=en_{\mathrm{free}\left(e\right)}\left(x,t\right)\mu_{0\left(e\right)}F\left(x,t\right)$$

(10) $$j_{c\left(h\right)}(x,t)=en_{\mathrm{free}\left(h\right)}\left(x,t\right)\mu_{0\left(h\right)}F\left(x,t\right)$$

Here, *j*_c(e)_ and *j*_c(h)_ represent the conduction current densities formed by electrons and holes in A m^−2^, respectively, while *µ*_0(e)_ and *µ*_0(h)_ are the carrier mobilities of electrons and holes controlled by shallow traps in m^2^ V^−1^ s^−1^, respectively.

Mobile charge carriers may be captured by deep traps during their migration, forming space charges. When trapped charges obtain enough energy, they can be released out of deep traps via thermal activation process. Traps in the CTMD model are assumed to be with single trap energy. Recombination occurs when electrons and holes encounter in the bulk of dielectric materials. Charge migration, trapping, detrapping, and recombination dynamic processes in the bulk obey conservation law and are described by the following four equations [18,24,34,35,36]:

(11) $$\frac{\partial n_{\mathrm{free}\left(e\right)}\left(x,t\right)}{\partial t}+\frac{1}{e}\frac{\partial j_{c\left(e\right)}\left(x,t\right)}{\partial t}=-\frac{n_{\mathrm{free}\left(e\right)}}{\tau_{\mathrm{tr}\left(e\right)}}\left(1-\frac{n_{\mathrm{trap}\left(e\right)}}{N_{T\left(e\right)}}\right)+\frac{n_{\mathrm{trap}\left(e\right)}}{\tau_{\mathrm{de}\left(e\right)}}-R_{e\mu ,h\mu }n_{\mathrm{free}\left(e\right)}n_{\mathrm{free}\left(h\right)}-R_{e\mu ,\mathrm{ht}}n_{\mathrm{free}\left(e\right)}n_{\mathrm{trap}\left(h\right)}$$

(12) $$\frac{\partial n_{\mathrm{trap}\left(e\right)}\left(x,t\right)}{\partial t}=\frac{n_{\mathrm{free}\left(e\right)}}{\tau_{\mathrm{tr}\left(e\right)}}\left(1-\frac{n_{\mathrm{trap}\left(e\right)}}{eN_{T\left(e\right)}}\right)-\frac{n_{\mathrm{trap}\left(e\right)}}{\tau_{\mathrm{de}\left(e\right)}}-R_{\mathrm{et},h\mu }n_{\mathrm{trap}\left(e\right)}n_{\mathrm{free}\left(h\right)}$$

(13) $$\frac{\partial n_{\mathrm{free}\left(h\right)}\left(x,t\right)}{\partial t}+\frac{1}{e}\frac{\partial j_{c\left(h\right)}\left(x,t\right)}{\partial t}=-\frac{n_{\mathrm{free}\left(h\right)}}{\tau_{\mathrm{tr}\left(h\right)}}\left(1-\frac{n_{\mathrm{trap}\left(h\right)}}{N_{T\left(h\right)}}\right)+\frac{n_{\mathrm{trap}\left(h\right)}}{\tau_{\mathrm{de}\left(h\right)}}-R_{e\mu ,h\mu }n_{\mathrm{free}\left(e\right)}n_{\mathrm{free}\left(h\right)}-R_{\mathrm{et},h\mu }n_{\mathrm{trap}\left(e\right)}n_{\mathrm{free}\left(h\right)}$$

(14) $$\frac{\partial n_{\mathrm{trap}\left(h\right)}\left(x,t\right)}{\partial t}=\frac{n_{\mathrm{free}\left(h\right)}}{\tau_{\mathrm{tr}\left(h\right)}}\left(1-\frac{n_{\mathrm{trap}\left(h\right)}}{N_{T\left(h\right)}}\right)-\frac{n_{\mathrm{trap}\left(h\right)}}{\tau_{\mathrm{de}\left(h\right)}}-R_{e\mu ,\mathrm{ht}}n_{\mathrm{free}\left(e\right)}n_{\mathrm{trap}\left(h\right)}$$

Here, *N*_T_ is the density of deep traps in m^−3^, while *R*_eµ,hμ_, *R*_et,ht_ and *R*_et,hμ_ are the recombination coefficients in m^3^C^−1^s^−1^. Based on the Langevin recombination model [39], recombination coefficient between free electrons and free holes can be expressed as, *R*_eμ,hμ_ = (*μ*_0(e)_ + *μ*_0(h)_)/*eε*_0_*ε*_r_. According to Shockley-Read-Hall model [40,41], trap-assisted recombination coefficients between free electrons and trapped holes and that between trapped-electron and free-hole can be expressed as, *R*_eμ,ht_ = *μ*_0(e)_/*eε*_0_*ε*_r_ and *R*_et,hμ_ = *μ*_0(h)_/*eε*_0_*ε*_r_, respectively. The units of *R*_eµ,hμ_, *R*_et,ht_ and *R*_et,hμ_ are all in m^3^C^−2^s^−1^.

Both molecular chains with dipole moment and those with occupied deep traps can move under the external electric field driven by electric forces [37,42,43]. As shown in Figure 6b, molecular chains with deep traps occupied by electrons will move toward the anode, while those with deep traps occupied by holes will move toward the cathode. The displacement of a molecular chain depends on the retention time of charges in trap centers which determines the duration of electric force acted on the molecular chain. The retention time of charges in deep traps is longer than that of charges in shallow traps, accordingly, the electric force acts a longer period on molecular chains with occupied deep traps. The velocity equation for the motion of a molecular chain with trapped charges is expressed as [37,42,43],

(15) $$d\lambda /dt=\mu_{\mathrm{mol}}F-\lambda /\tau_{\mathrm{mol}}$$

Here, *λ* is the displacement of a molecular chain in m, while *μ*_mol_ is the mobility of molecular chains in m^2^V^−1^s^−1^, which is determined by the carrier mobility hopping among shallow traps and the trapping of deep traps, namely *μ*_mol_ = *μ*_0_*τ*_tr_/(*τ*_tr_ + *τ*_de_). Furthermore, *τ*_mol_ is the relaxation time constant of molecular chains in s, which equals the retention time of charges in deep traps, *τ*_mol_ = *τ*_0_exp(*E*_T_/*k*_B_*T*).

The displacement of molecular chains with occupied deep traps will enlarge the local free volume in nanocomposite dielectric materials. When the energy of electrons gained from electric field in the enlarged free volume exceeds a threshold value, the energy of deep traps, electrical breakdown would be triggered [34,44]. Since the displacement of a molecular chain with occupied deep traps would determine the electrical breakdown process, in the following numerical simulations we focus on the motion of molecular chains with deep traps.

### 4.2. Parameters and Numerical Computation Methods

The temperature and thickness of samples are the same as those in experiments, which are 33 °C, and 100 µm, respectively. The relative permittivity is 2.3 measured by broadband frequency spectrometer (Concept 80 Novocontrol, Germany) at a frequency of 1 MHz at room temperature. In addition, the carrier mobilities of electrons and holes controlled by shallow traps both are assumed to be 1 × 10^−13^ m^2^V^−1^s^−1^ [24,45]. The effective carrier mobility will be modulated by the trapping and detrapping dynamics of deep traps. The energy and density of deep traps and the relaxation time constant of charges in deep traps were characterized by TSDC apparatus for PP/Al_2_O_3_ nanocomposite dielectric materials and their results shown in Figure 2b are used in simulations. The charge injection from metal electrode into dielectric materials is influenced by surface states such as surface traps and dipoles of dielectrics, which has been evidenced by experiments [32,46] and calculation results of density functional theory [47,48]. It was proposed that charges on metal electrode may transfer to deep surface traps and then are injected into the conduction band via thermal-field activation [47,48]. Consequently, the effective injection barriers for electrons and holes are assumed to be the same as the energy of deep traps, which are 0.86 eV. The trapping coefficients of deep traps which is the reciprocal of mean time period of mobile charge carriers between two trapping events are assumed to be 0.085 s^−1^ [24,45]. 

Nanocomposite dielectric materials with the thickness of 100 μm is divided into 500 parts, namely, each part Δ*x* is 0.2 μm. The computation time step Δ*t* is set as 1 ms. High order accuracy and stable Runge-Kutta Discontinuous Galerkin method was utilized to solve the charge continuity Equations (11) to (14) [49,50,51]. Poisson’s Equation (6) was solved numerically by Finite Element method. Computed by the CTMD model, dc electrical breakdown strength and distributions of space charges, electric fields, and molecular displacement can be obtained.

### 4.3. Simulation Results and Discussion

**Figure 7:**
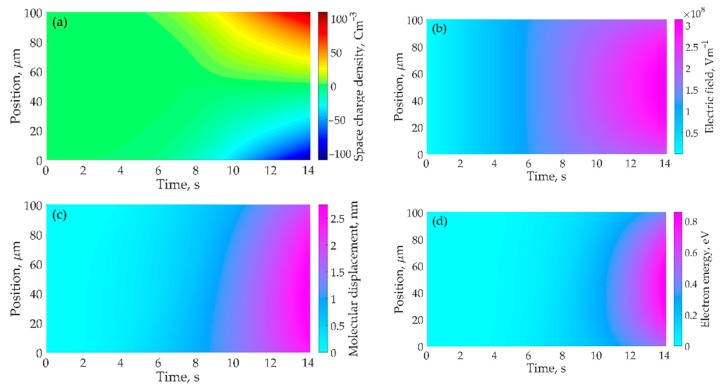
Numerical results of space charge distribution profiles (**a**), electric field distribution profiles (**b**), molecular displacement distribution profiles (**c**), and electron energy distribution profiles (**d**) as a function of time in neat PP dielectric material.

Figure 7a demonstrates the distributions of space charges accumulated in neat PP dielectric materials at various times after the application of a ramp voltage. The cathode and anode are at the positions of *x* = 0 and *x* = 100 μm, respectively. Under high electric fields, homo space charges accumulate near the interfaces between dielectric material and its electrodes. With an increase in time, the voltage applied on samples increases with time at a set ramping rate, namely *V*_appl_ = *k*_ramp_*t*_ramp_*.* Charges are injected into the dielectric material continuously, thus homo space charges accumulated monotonically with time. For example, the space charge density near the cathode is 2.6 Cm^−3^ at 5 s, 37.5 Cm^−3^ at 10 s, and 107.6 C m^−3^ at 14 s for neat PP dielectric material. Space charges are gradually decreased from the interfaces near electrodes to the middle of bulk, and a large amount of space charges accumulate at the interfaces near electrodes. Figure 7b shows the distributions of electric fields in neat PP dielectric materials at various times after the application of a ramp voltage. Electric fields tend to be strengthened as a result of an increase in time or applied voltage. The homo space charges accumulated at the interfaces build up an electric field in an inverse direction with applied electric field according to Poisson’s equation, resulting in that the electric fields gradually increase from the interfaces between dielectric material and its electrodes to the middle of bulk. Therefore, the electric field near the interfaces are reduced, while electric fields in the middle of the bulk are enhanced. The electric field in the middle of neat PP material is 99.6 kV mm^−1^ at 5 s, 212.2 kV mm^−1^ at 10 s, and 310.6 kV mm^−1^ at 14 s. The simulation results are consistent with experimental results of space charge and electric field distributions in PP dielectric material [9,19,20,23].

According to the motion dynamics of molecular chains with occupied deep traps, namely Equation (15), molecular displacement increases with increasing the electric field and time as shown in Figure 7c. When the first term in the right part of Equation (15) dominates the motion dynamics of molecular chains, the Equation can be simplified to be d*λ*/d*t* = *μ*_mol_*F*. If we do not consider the distortion of electric field caused by space charges, we can obtain the time dependent molecular displacement *λ* = (*k*_ramp_*μ*_mol_/2*d*)*t*^2^, by replacing *F* by *k*_ramp_*t*/*d* and considering the initial condition *λ*(*t* = 0) = 0. It indicates that *λ* is proportional to the square of time. When the distortion of electric field is considered, we will obtain similar distributions of molecular displacement to those of electric field. The maximum local *λ* is 0.34 nm at 5 s, 1.33 nm at 10 s, and 2.71 nm at 14 s. As some molecular chains with occupied deep traps move towards electrodes driven by electric force, the free volume around the molecular chains would be enlarged. It is assumed that the length of local free volume equals to the displacement of a molecular chain with occupied deep traps. Mobile charges migrate in the free volume can gain energy from electric field, and their energy *w* is determined by both the local electric field *F* and the length of free volume *λ*, namely *w* = *eFλ*. The distribution of electron energy gain in free volume is shown in Figure 7d. The electron energy gain in the middle of the neat PP material is 0.03 eV at 5 s, 0.27 eV at 10 s, and 0.83 eV at 14 s. When the maximum energy of electrons gained from electric field in a free volume exceeds the energy of deep traps, electrons will transport freely in the dielectric material without trapping. The local current density would be high enough to trigger electrical breakdown inside the material. In addition, the electrical breakdown is initiated at the position with the highest electric field.

**Figure 8:**
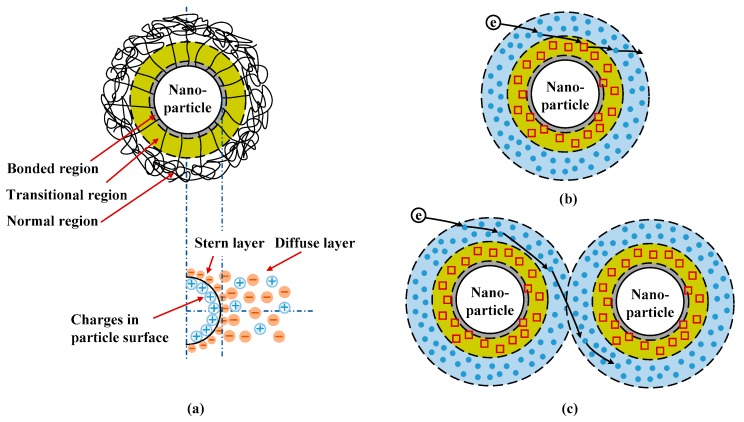
Schematic diagram of interfacial region around nanoparticles in PP/Al_2_O_3_ nanocomposites; (**a**) multi-region model of interfacial region, (**b**) charge carrier transport through an isolated interfacial region, and (**c**) charge carrier transport through overlapped interfacial regions.

Incorporating nanofillers into polymer can form interfacial regions or interaction zones. It has been suggested that these interfacial regions can vary the trap properties, which will change charge transport, the accumulation of space charges, and electrical breakdown properties. Interfacial regions exist between nanoparticles and polymer matrix has been evidenced by the results of differential scanning calorimetry and transmission electron microscopy [52], images of atomic force microscopy and electrostatic force microscopy [53], dielectric spectroscopy [54], and so on. The mesoscopic structure of interfacial regions is influenced by the molecular structure, polarity, cohesion energy of polymers, and by the type, polarity, crystal structure, surface functionalization of nanofillers [8,14]. From the viewpoint of mesoscopic structures, interfacial regions around nanoparticles can generally be divided into several sub-regions, which are bonded, transitional, and normal regions as shown in Figure 8 [14]. When unsaturated bonds, hydrogen bonds, and other organic groups are introduced on the surface of nanoparticles, strong interaction between nanoparticles and polymeric matrix can be formed via covalent and hydrogen bonds. A tight coupling region or bonded region is generated around nanoparticles with a thickness of about one to several nm. The interaction intensity between nanoparticles and polymer matrix in bonded region is affected by the surface properties and polarity of nanoparticles as well as the characteristic of polymer. The treatment of nanoparticles by silane coupling agent can reinforce the interaction intensity in bonded region. Moreover, a transitional region is formed by regular arranged molecular chains extended from the bonded region to tens of nm. The bonded and transitional regions can change the morphology and crystallinity of polymer nanocomposites. A normal region exists in the area extended from the transitional region to tens of nm, and it has similar properties to polymer matrix [14].

**Figure 9:**
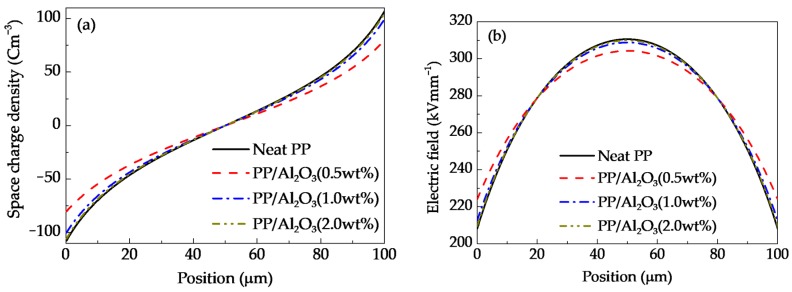
Distributions of space charge density (**a**) and electric field (**b**) at the application time of 14 s (namely *V*_appl_ = 28 kV) for nanocomposite dielectric materials at various nanofiller loadings.

It was suggested that deep traps may be introduced in the bonded and transitional regions and shallow traps may exist in the normal region, as shown in Figure 8b [6,8,14,15,16]. It was indicated that deep traps dominate the short-term electrical properties of nanocomposites at relatively low nanofiller loadings, while shallow traps play an important role in the long-term electrical properties at relatively high nanofiller loadings [6,8,14,15,16]. At relatively lower nanofiller loadings, isolated interfacial regions are formed in polymer nanocomposite dielectrics. Deep traps in the bonded and transitional regions can capture mobile charges effectively so that the migration of charge carriers is decelerated. Moreover, deep traps formed in isolated interfacial regions near the surfaces of dielectric materials can hinder the charge injections from electrodes into nanocomposite dielectrics. Figure 9a demonstrates the distribution profiles of space charges accumulated in nanocomposite dielectric materials with nanofiller loadings of 0 wt %, 0.5 wt %, 1 wt %, and 2 wt % at the application time of 14 s (namely *V*_appl_ = 28 kV). The space charge density decreases firstly and then increases with an increase in nanofiller loading. The space charge density near the interface between dielectric material and anode is 107.0 Cm^−3^ at the loading of 0 wt %, 79.9 Cm^−3^ at 0.5 wt %, 99.6 Cm^−3^ at 1.0 wt %, and 105.2 Cm^−3^ at 2.0 wt %. According to Poisson’s Equation (6), the more space charges are accumulated inside dielectric materials, the more severely electric field will be distorted. Figure 9b shows the distributions of electric fields in nanocomposite dielectric materials with nanofiller loadings of 0 wt %, 0.5 wt %, 1 wt %, and 2 wt % at the application voltage of 28 kV. The distortion coefficient that is defined by the maximum electric field divided by the minimum one is 1.49 at the loading of 0 wt %, 1.36 at 0.5 wt %, 1.45 at 1.0 wt %, and 1.48 at 2.0 wt %. The simulated distributions of space charges and electric fields changed by nanodoping is similar to previous findings [9,20].

**Figure 10:**
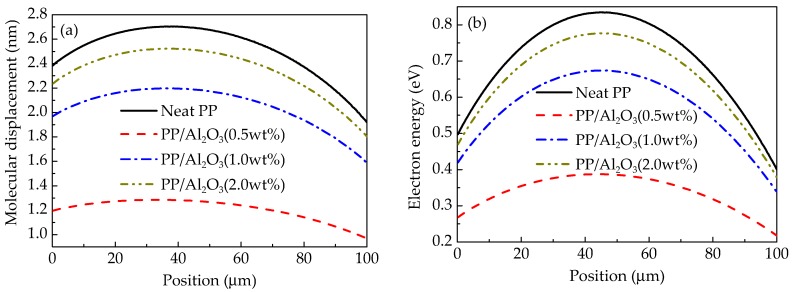
Distributions of molecular displacement (**a**) and electron energy (**b**) at the application time of 14 s (namely *V*_appl_ = 28 kV) for nanocomposite dielectric materials at various nanofiller loadings.

The molecular chain with occupied deep traps will move under the electric field. Figure 10a shows the distributions of molecular chain displacement in nanocomposite dielectric materials with nanofiller loadings of 0 wt %, 0.5 wt %, 1 wt %, and 2 wt % at elapsed time of 14 s after the application of a ramp voltage. The maximum displacements of molecular chains with occupied deep electron traps are 2.71, 1.29, 2.20, and 2.52 nm in PP nanocomposite dielectric materials with nanofiller loadings of 0 wt %, 0.5 wt %, 1 wt %, and 2 wt % respectively, after applying a ramp voltage for 14 s. The motion of molecular chains with occupied deep traps is modulated by the intermolecular interactions, which is changed by the variation of nanofiller loading [55]. At low nanofiller loadings, the interactions between nanoparticles and polymer matrix increase the friction among molecular chains. Accordingly, the molecular mobility decreases or the relaxation time of molecular chains increases with an increase in nanofiller loading [55], as demonstrated in Figure 6b. When the nanofiller loading exceeds a critic value, the mean distance between two neighbor nanoparticles is very small and a polymer-mediated nanoparticle network will be formed. The molecular chain dynamics may transform from polymer-like to gel-like. The relaxation time of molecular chains decreases with nanofiller loading above a critic value [55]. Consequently, the motion velocity of molecular chains decreases firstly and then increases with the increase in nanofiller loading.

The molecular chain displacement may result in the increase in the length of free volume in dielectric materials [34,56,57]. It is assumed that the length of free volume is very small in the initial state for PP nanocomposite dielectric materials at room temperature. Therefore, the length of free volume *λ* is equal to the displacement value of molecular chains under the applied electric field. The molecular chain displacement or the length of free volume increase with the applied voltage increases. The increases in both local electric field and molecular chain displacement lead to the increase in the energy of electrons gained from electric field in the free volume. Figure 10b shows a comparison of electron energy gain distributions in PP nanocomposite dielectric materials. The energy of electrons gained from electric field in free volume enlarged by molecular chain displacement decreases firstly and then increases with an increase in nanofiller loading. The maximum energies of electrons in PP nanocomposite dielectric materials with nanofiller loadings of 0 wt %, 0.5 wt %, 1 wt %, and 2 wt % after applying a ramp voltage for 14 s are 0.83, 0.39, 0.67, and 0.78 eV respectively.

**Figure 11:**
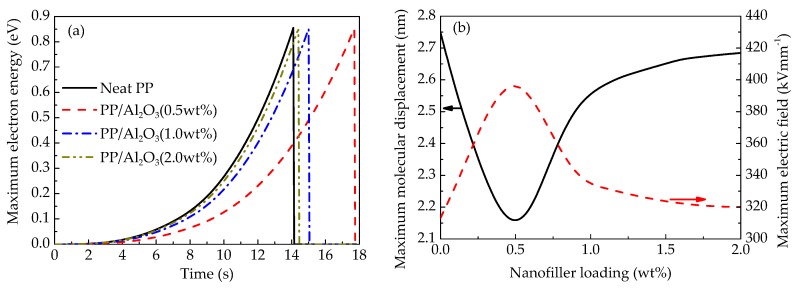
Numerical results of time-dependent maximum electron energy in nanocomposites at various nanofiller loadings (**a**) and maximum molecular displacement and maximum local electric field as a function of nanofiller loading at pre-breakdown (**b**).

Figure 11a demonstrates the maximum energy of electrons gained from electric field in free volume enlarged by the displacement of molecular chains of PP nanocomposites at various nanofiller loadings according to the calculations by the CTMD model. Electron energy gain is determined by both electric field and the length of free volume. The electric field in nanocomposites increases with the application time of ramp voltage and the maximum local electric field is enhanced by the accumulation of homo space charges. Moreover, the free volume is enlarged by the displacement of molecular chains with occupied deep traps. In other words, the length of free volume, the integration of the product of electric field and the effective mobility of molecular chain over time, increases with time. The effects of both the enhanced electric field and the enlarged free volume result in the increase in the energy gain of electrons. When the maximum electron energy *w*_max_ = (*eFλ*)_max_ exceeds the trap energy level *E_T_*, mobile charge carriers will migrate in dielectric materials without trapping by deep traps, resulting in the multiplication of local currents and the sudden rise of local temperature. Eventually, electrical breakdown would be triggered [31,44]. Since the motion of molecular chains is restricted by nanoparticles in nanocomposites with relatively lower loadings, the molecular displacement is smaller than that of neat PP as shown in Figure 11b. It indicates that the local free volume is enlarged slower in lightly doped nanocomposites than that in neat PP. Nevertheless, when nanofiller loading is higher than 0.5 wt %, the molecular displacement as well as the local free volume increase with increasing the nanofiller loading. Since dc electrical breakdown is triggered when the maximum electron energy, the product of local free volume and electric field, exceeds a critical value, the maximum electric field at pre-breakdown is inversely proportional to the free volume or molecular displacement as demonstrated in Figure 11b. Therefore, the breakdown electric field increases firstly and then decreases with an increase in nanofiller loading.

**Figure 12:**
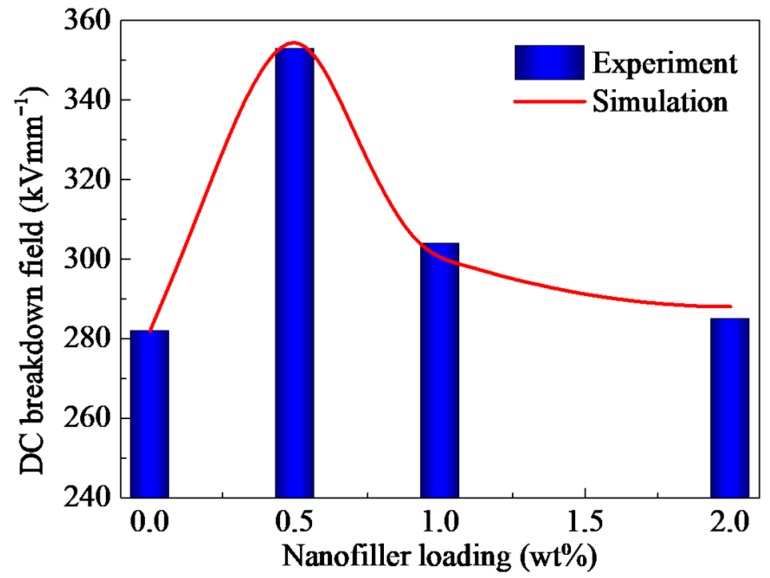
Comparison of experimental and simulation results of dc electrical breakdown field.

Figure 12 shows the comparison of characteristic electrical breakdown strength of experimental and simulation results. It is found that simulation results are in a good agreement with experimental results as shown in Figure 4. The calculated results of dc electrical breakdown strength are 282.1, 358.1, 324.7, and 292.4 kV mm^−1^ for PP/Al_2_O_3_ nanocomposite dielectric materials with nanofiller loadings of 0 wt %, 0.5 wt %, 1 wt %, and 2 wt %, respectively. It indicates that the CTMD model consisting dynamics of charge transport and molecular chain displacement can well interpret the nanofiller loading dependent dc electrical breakdown experiments of PP nanocomposite dielectrics.

## 5. Conclusions

The influencing mechanism of nanodoping on dc electrical breakdown properties of PP/Al_2_O_3_ nanocomposite dielectric materials were investigated by experiments and simulations. Nanocomposite dielectric materials with various nanofiller loadings were prepared by melt-blending and hot-pressing methods and nanofillers are homogeneously dispersed in the matrix as indicated in SEM images. Experiments of thermally stimulated current, surface potential decay, and dc electrical breakdown show that incorporating a small amount of nanofillers into polymer matrix increases the retention time of deep trapped charges and hinders the charge transport in nanocomposite dielectrics, resulting in the enhancement of dc electrical breakdown field. Based on the experimental results, a model considering charge transport dynamics and the displacement of molecular chains with occupied deep traps was utilized to simulate the dc electrical breakdown behavior of nanocomposite dielectrics. When the electron energy gain from electric field in free volume enlarged by the molecular displacement exceeds the potential barrier, electrical breakdown would be triggered. It was found that the electrical breakdown field increases firstly and then decreases with an increase in nanofiller loading. At relatively low loadings isolated interfacial regions are formed in nanocomposite dielectrics, resulting in the increases in the retention time of deep trapped charges and the interaction between molecular chains. Accordingly, the transport of charges and the displacement of molecular chains with occupied deep traps are hindered, which can enhance the electrical breakdown strength. At relatively high loadings interfacial regions may overlap in nanocomposite dielectrics so that the retention time of deep trapped charges decreases. The molecular chain dynamics may transform from polymer-like to gel-like and the interaction between molecular chains may be weakened, causing the acceleration of molecular motion driven by electric force and the decrease in electrical breakdown strength. It was concluded that the changes in the charge transport and molecular displacement characteristics affected by nanodoping may be the influencing mechanism of dc electrical breakdown properties of PP nanocomposite dielectrics.
